# How Reasons Make Law

**DOI:** 10.1093/ojls/gqad026

**Published:** 2023-12-13

**Authors:** Angelo Ryu

**Keywords:** anti-positivism, natural law, philosophy of law, political obligation

## Abstract

According to legal anti-positivism, legal duties are just a subset of our moral duties. Not every moral duty, though, is legal. So what else is needed? This article develops a theory of how moral duties come to be law, which I call the constitutive reasons account. Among our moral reasons are legal reasons—and those reasons make moral duties into legal duties. So the law consists of moral duties which have, as one of their underlying reasons, a legal reason. Such legal reasons arise from a relationship with the body for which it is the law of. The legal reasons in America, then, are the moral reasons flowing from a relationship with the United States. These reasons include consent, democracy, association and fair play. They are law’s constitutive reasons. By looking for them, we can better explain why some moral duties form part of the law, while others do not.

## 1. Introduction

Some say our legal duties are also moral duties.^1^ The law is therefore part of the general moral landscape. It forms a subset of the broader moral picture. I shall call this view anti-positivism.

Whatever its merits, anti-positivism faces a serious problem. Note what anti-positivists do not say: they do not implausibly claim all moral duties are legal. All legal duties might be moral, but not the other way around. Hence, we can intelligibly say ‘this is not yet illegal, but it should be because it is a grave moral wrong’. This brings up a demarcation problem, for we need to know which moral duties are legal and which are not. The key question is this: what additional feature must moral duties possess before they are law?

Some anti-positivists are sceptical of the stakes of this question.^2^ They already think legal duties are moral duties. What, then, motivates the search for a strict line between law and the rest of morality?

The motivation, I think, lies in our experience. Often we refer to our rights and duties under law. In doing so, we rely on our intuitive grasp of law as a distinct set of normative incidents. It is something legislators can make, judges can apply, lawyers can argue about and students can study. But not every moral duty is legislated, judicially applied, relied on by lawyers or studied in law schools. Drawing this distinction is a fundamental feature of legal practice. To explain it, anti-positivists  need to show what sets law apart.^3^ Currently, there are two main proposals.

Greenberg offers one: that legal duties are those moral duties which legal institutions cause in the legally proper way.^4^ This is a causal pedigree approach to demarcation. The law consists of changes which legal institutions make to our moral situation.

At least on some readings, Dworkin disagrees.^5^ He says, instead, that legal duties are those moral duties which are enforceable in court. This is a judicial enforceability approach to demarcation. Sometimes we can require the court, on demand, to wield its coercive power to ensure the satisfaction of our moral rights. The law consists of these circumstances.

We can put the difference between these two approaches in temporal terms. Greenberg looks to the past. He tells a historical story of how the change to our moral duties arose. Conversely, Dworkin looks to the future. He tells a prospective story of what should occur if we invoke our moral rights.^6^

Take the legal duty to drive on the left side of the road. For an anti-positivist, this is a moral duty. Driving on the right would be morally wrong. But why is it a legal duty? Greenberg says it is because of how the traffic duty came about. It arose because the legislature caused us to converge on a set of expectations. Now, we expect the other cars to drive in the left lane. And that gives us a duty to drive in the left lane, too. Since we can causally trace the duty back to what the legislature did, it is law. Dworkin gives a different answer. He says it is because of what will occur if we breach the duty. Such a breach would be morally wrong. But not just that—a court could justifiably impose coercive sanctions in response. This possibility is why the duty is law.

Both approaches have serious flaws, which I address later. For now, I want to float a third possibility. As we saw, these approaches look to either the past or future. *But what about the present?* That is, we could start with the nature of the legal duties themselves—not their origin story or their significance in the courtroom.

Let us return to the traffic example, and this time take a closer look at what underlies the duty to drive in the left lane. Recall that, for anti-positivists, it is a moral duty. Here I assume moral duties consist of reasons against φ-ing, which together render φ-ing impermissible. If so, moral duties are constituted, at least partly, of reasons.

This allows for an alternative approach. Legal duties are those moral duties partly composed of a legal reason. Driving on the right lane may be morally impermissible for many reasons. Among those reasons, however, is a legal reason—and that makes the traffic duty form part of the law. Call this the constitutive reasons account.

For it to succeed, we must work out the legal reasons. I think those reasons are moral considerations, so they cannot be in contrast to morality. Hence, my account is anti-positivist. But now it seems we have traded one problem for another. Previously, we wanted to know why the law consists of some moral duties, but not all. This approach, however, throws up a different problem: what makes some moral reasons, but only some, legal reasons?

Here is my answer. Legal reasons are those moral reasons which arise from, and apply within, a relationship to the relevant law-having body. This is ecumenical in two ways. First, it allows a plural set of moral considerations to count as a legal reason. Any reason we only owe within a relationship is, in principle, a possible legal reason. Second, it allows for different kinds of law. Municipal law concerns the reasons which flow from a relationship to the state, canon law from a relationship to the church, international law from a relationship to the world community, and so on.

To bring things together, let us return to the moral duty to drive in the left lane. Among the ways its breach would be wrong is this legal reason: the consideration of fair play which flows from our participation in a state-run system of traffic co-ordination for mutual benefit. Since this reason only applies within our relationship with an aspect of the state, it is a legal reason. So its presence makes the moral duty a legal duty, too.^7^

The article is laid out as follows. The second section engages critically with Greenberg’s causal pedigree approach. The third section does the same for Dworkin’s judicial enforceability approach. Together, they reveal issues which an alternative approach could address. The fourth and fifth sections introduce the constitutive reasons account. The sixth section shows why it is more attractive than the alternatives. The seventh section responds to objections. A brief conclusion follows.

## 2. The Causal Pedigree Approach

For Greenberg, the law consists of changes which legal institutions cause to our moral situation. So law is the moral impact of legal institutions. Not every change, however, is part of the law. As Greenberg recognises, this would be overinclusive. Suppose a legal institution acts in an evil way. For instance, the executive targets specific groups for detention in concentration camps. This causes a significant change in the state of affairs. The change generates a moral duty to resist. We ought to do what we can to hinder the executive’s ability to carry out this plan. But this moral duty is not plausibly a legal duty. Certainly, the moral duty to rescue the Nazi regime’s victims was not part of Nazi law.

In response, Greenberg restricts his theory to those changes legal institutions cause in the legally proper way.^8^ For him, legal institutions which worsen the moral situation do not pass this test. The causality is deviant because legal institutions exist to improve the moral situation. When legal institutions worsen the profile, thereby causing duties to resist, the directionality is flipped. Compliance aims to halt, indeed reverse, the change to the moral situation.

Here is the problem.^9^ As it turns out, it is not so easy to know whether the ensuing moral duty is one of resistance. An intuitive strategy is to rely on the legal institution’s intention, or alternatively the content of what that legal institution produced. That is, we might learn the legislature intended a grave moral wrong. Or what the legislature produced—the statute—might, on its face, require a grave moral wrong. Either possibility allows us to identify the moral wrong with what the legal institution tried to do. Only then can we say the moral duty to prevent that wrong from arising is one of opposition to the legal institution’s actions.

But Greenberg disclaims resort to either intention or the linguistic meaning of legislation.^10^ How, then, can we identify which moral duties are of resistance? A possible view is that such duties arise when we ought to resist the law. But this option is not available to Greenberg, for, as an anti-positivist, he is committed to the view that legal duties are a subset of moral duties. And here the legal institution acts so wrongly that not only does it fail to require us to comply, but we are morally required to engage in active resistance. So the thing we must resist cannot, for Greenberg, be a legal duty. Nor, as we saw, can it be what the legal institution intends, or what the statutory meaning requires.

What is left? Recall we need to ascribe a possible state of affairs—which we are morally required to prevent from ever occurring—to what the legislature did. To do so, it seems Greenberg must retreat to a probabilistic view. That is, a legal institution is causally responsible for a moral wrong when it, by acting, increases the probability of that wrong arising. For instance, by enacting a statute which calls for concentration camps, the legislature makes it more likely the moral wrong of herding people to such camps occurs. We have a moral duty to prevent this from occurring. The duty is one of opposition to the legislature since the legislature made it more likely the wrong will occur. This allows Greenberg to deny legal status to this moral duty.

But the probabilistic view is implausible. Suppose the legislature declares war on Country X. To that end, it seeks to conscript able-bodied adults into the military. This, let us say, worsens the moral profile. It would be better had the legislature not declared war. Indeed, the case for war is so weak that, in ordinary circumstances, the legislature would *fail* to impose a duty on us to join the military. However, by declaring war, the legislature makes it more probable that Country X will commit serious moral atrocities. To stop it, able-bodied adults are thereby under a duty to fight. Intuitively, this duty is both moral and legal. And so, by joining the military, able-bodied adults comply with their legal duty. But the probabilistic account cannot explain this. From its perspective, this is a duty to resist the grave moral wrong of Country X committing atrocities. And that is a wrong which the legislature’s act made more likely to occur. It therefore arises in a legally improper way. Hence, there is no legal duty to join the military.

Greenberg faces other problems, too. Consider a legislature which acts to impose a tax. Because of this, my friends need to fill out complicated paperwork. Since they are struggling, I promise to help. I thereby come under a moral, promissory duty. It is caused, in part, by what the legislature did. But it is not a legal duty.

Once again, Greenberg says the duty does not arise in the legally proper way,^11^ for these duties are too far downstream—that is, too remote—from the legal institution’s act. So the causal process is legally improper. But this, like his attempt to exclude duties to resist from law, does not work. To see this, consider what remoteness might mean.

There are two intuitive possibilities. First, consequences are too remote insofar as they are unforeseeable. But it may well be readily foreseeable that, by imposing a tax, people will promise others to help with the paperwork necessary to pay that tax. Second, consequences are too remote insofar as the acts of others ‘break the chain of causation’. On this view, the legal institution may have led to my having a promissory duty to assist. Since I chose to make that promise, however, it is too remote, for my free choice has broken the causal chain. But this is of no help, for we could simply change the facts to make my duty to assist non-consensual. Suppose my duty to help fill out the tax form is owed not to my friends by virtue of a promise, but to my parents by virtue of our relationship. On these facts, I do not choose to come under the moral duty to assist. So I have not broken the chain. Nonetheless, my duty is not legal.

To sum up, Greenberg seeks to address these potential cases of overinclusion by adding a caveat to his theory. He says law only consists of changes which legal institutions cause in the ‘legally proper way’. The worry is this formulation simply reflects an intuition that these duties are not law.^12^ If that is so, the caveat is just an empty label. It offers no positive explanation for the intuition.

## 3. The Judicial Enforceability Approach

For Dworkin, the law consists of moral duties which are judicially enforceable. To have a legal right, as opposed to just a moral right, is to be entitled to have a court vindicate that right. If my right to £10 is legal, failing to respect my right is not just morally wrong. I am entitled to demand the money in court. And the court must then direct the state’s coercive authority to *make* you pay £10.

This approach is underinclusive of law.^13^ Some legal duties fall prey to a procedural defect, like the expiration of a limitation period. Say the victim waits too long to bring a claim. Here there is a strong intuition, supported by legal practice, that the victim continues to have a right as a matter of substantive law. The victim is just procedurally barred from enforcement. Similarly, the rules of evidence could stop a plaintiff from proving the allegations in court. In practice, this leads to unenforceability. But suppose the allegation is factually true. It just cannot be proven in court. Here, you might, once again, think the plaintiff possesses a substantive legal right.

The problems go beyond procedure. As a matter of substance, the claim might concern a non-justiciable subject matter.^14^ If so, the plaintiff possesses a legal right as a first-order matter. But the court, as a second-order matter, lacks the power to adjudicate. So the legal right is unenforceable. Yet, dismissal has nothing to do with the merits—that is, whether the plaintiff has the legal right she asserts.

Given this, you may wish to revise the view. Perhaps a moral duty is legal if it is enforceable in court, or if it is unenforceable because a separate legal rule either requires or permits a court not to enforce it. Since procedural limits and the justiciability doctrines are legal rules, this revised approach can correctly identify the duties they render unenforceable as law.

But this remains seriously overinclusive. For instance, English courts cannot recognise new criminal offences in common law.^15^ This is a legal duty which prevents courts from enforcing moral duties not found in legislation. Yet, we would not describe all these unenforceable moral duties as legal duties. In response, you could say this legal rule just reflects the moral position. With or without the rule, it would be wrong for courts to recognise new common law offences. But this cannot help Dworkin, for we could say the same thing about non-justiciability. Given the lack of judicial capacity, it would be wrong for courts to enforce a duty in a non-justiciable area. Those non-justiciable duties can still be legal duties, however.

## 4. The Constitutive Reasons Approach

We have dwelt for too long on the shortcomings of other approaches. It is now time to see whether we can do better.

I think we can, by focusing on the reasons which make up duties. Take our duty not to steal. What explains this duty? Well, theft is wrong for all sorts of reasons. It deprives the victim of a valuable interest. It manifests disrespect towards her. It causes her distress. And so on. These are all plausible considerations which count against stealing. Together, the force of those reasons render theft impermissible.

Suppose a state which prohibits theft *adds* to the reasons not to steal. Afterwards, there would just be one more reason, among many, against it. The presence of this additional reason sets this moral duty apart from others. This raises a tempting possibility. We might say a legal duty is just a moral duty which has, as one of its underlying reasons, a legal reason.^16^

Such a view merely requires the *presence* of this reason. This says nothing about its relative importance. There are two possibilities. First, the legal reason could be decisive for the duty to arise. The duty to pay taxes is a good example. Without the law, the duty would not arise. Second, the legal reason could be unnecessary for the duty. Indeed, the force of some moral duties is so overwhelming that the addition of reasons, including legal reasons, seems insignificant.^17^ Take, for instance, the duty not to murder. So long as one of the reasons composing that duty is legal, however, it forms part of the law.^18^

This shifts much of the explanatory burden from the duty to the reasons underlying that duty. A moral duty is legal when one of the reasons for complying with that duty is of the right sort. So everything hinges on the following question: what makes a reason a legal reason?

That is what the next section seeks to answer.

## 5. Legal Reasons

Consider the duty to drive in the left lane. Two distinct set of considerations could support this duty. First, because it promotes road safety. Needlessly injuring another is wrong, and sticking to the left lane reduces the risk of that occurring. Second, because we agreed to drive in the left. Or it is what a democratically elected body chose. Or it is a fair price to pay for the benefits we obtain from co-operatively driving on the road.

That latter set encompasses a diverse range of considerations. These include consent, democracy and fair play.^19^ Among others, they are what I call ‘legal reasons’. Now, you might doubt whether they all, in fact, support the traffic duty. Little turns on this. The traffic example is only illustrative. What matters is two things. First, these considerations sometimes count in favour of a legal duty, even if you reject their salience in the traffic context. Second, something unites these reasons as a single category, which then distinguishes it from our general reason not to injure others.

To be clear, I will not offer a sustained defence of how these particular considerations support legal duties. Their precise grounds—how they support the moral duties we have, so far as they do—is not something I address. All I want to establish is that these considerations are plausible candidates for the set of legal reasons. You may disagree with the precise picture, but I hope to show how they could support legal duties.

### A. Fair Play

At this point, I want to focus on a particular candidate for a legal reason: fair play. With this discussion I hope to clarify the kind of reasons I have in mind, and how they might bear on the legal duties we possess.

To achieve a common benefit, we sometimes need to work together. An obvious example might be a football team, which must co-ordinate to win. And winning, let us say, is beneficial to all members of that team. Being in a position to win, though, is not easy. It requires hard work, like gruelling practices. This gives rise to the intuition underlying fair play. One should not benefit from the hard work of one’s teammates if one does not put in the requisite work oneself. Doing so takes advantage of the efforts of others—and that is unfair. So there is reason to avoid taking a free ride.^20^

Many question the breadth of this consideration. Nozick, for instance, asks us to consider a neighbourhood association which operates an entertainment scheme.^21^ The group assigns each neighbour a day she is responsible for providing entertainment. Suppose the entertainment benefits each neighbour. Given this, must the neighbours entertain on their assigned day? Now, there are many ways to respond, but here I consider two.

First, we could distinguish a reason to entertain from a duty to do so. That is, we could accept the neighbours have reason to contribute, given the benefits they enjoy. But the benefit is not so important as to generate a duty.^22^ We could assess this importance in both objective and subjective terms. The entertainment may not objectively be too important. Alternatively, the neighbours may subjectively prefer other things over entertainment.

Second, we could distinguish passive receipt of benefits from active participation in the beneficial scheme.^23^ The members of a sports team are actively involved in the joint activity. They are not just bystanders who benefit from seeing the team play; in an important sense, they *are* the team. Similarly, it may not be enough for the neighbours to enjoy the entertainment. Perhaps they must participate in the neighbourhood association before coming under a duty to contribute.

Once we keep these limits in mind, it becomes easier to see how fair play reasons could arise. Those who actively participate in co-operative enterprises for a substantial mutual benefit ought to bear the reciprocal burdens.^24^

### B. Special Reasons

You may worry my account is arbitrary. What explains treating fair play as part of a distinctive set of reasons? Perhaps I have simply selected a kind of moral consideration from a grab bag of reasons. If so, the ensuing account would be objectionable as *ad hoc*. It may get us closer to our intuitions about which moral duties are legal, but at the cost of explanatory power.

It is good, then, that something is distinctive about fair play, alongside other considerations like consent and democracy. Some reasons apply to us all, by virtue of our being human. And we owe the duties they support to everyone else, by virtue of their being human. These are general reasons. Other reasons, however, only apply to those in particular relationships. And we only owe the duties they support to the other members of that particular relationship. These are special reasons.^25^

Consider the duty not to needlessly cause injury to others. We owe this to everyone else. It does not require us to be in a relationship, other than in the possible sense in which we are all members of a moral community. So the reason which supports this duty—the disvalue of injury—is a general reason.

Now consider the duty to do what I agree to do. Plausibly, I only come under the duty once I enter the agreement. The agreement forms a distinct relationship between me and you. Further, I owe the duty to respect that agreement to you, but not others. So the reason which supports this duty is a special reason. The same applies to the other considerations we addressed. The democracy-based reason only applies to members of a democracy, and we only owe the duties it supports to members of that democracy. And so on.

Put another way, special reasons arise, and are relevant to, the limited domain of a relationship. In the context of law, however, one particular relationship looms large: how we stand with respect to the state. For municipal law—the law of states, like American law—is surely of central significance to any account of law. So I want to start by addressing how my approach explains this important species of law.

Again, legal reasons are distinctive because they are special reasons. They only arise from, and apply within, particular relationships. In the context of municipal law, the relevant relationship is our relationship to the state. Within this domain, our legal reasons are the reasons which arise from our relationship with the state, or at least an aspect of the state. So the reasons which arise from our relationship to the United States explain why some of our moral duties form part of American law. Here, I understand ‘relationship’ quite broadly. This expansive understanding allows my account to encompass the kinds of considerations I have previously mooted.

First, consent. By agreeing to have certain duties apply to me, I enter into a consensual relationship with the state—and that may entail a relational duty to keep my word.^26^ Second, democracy. I share a relationship of citizenship with my fellow compatriots, and that may entail a relational duty to respect their views. Third, fair play. By participating in beneficial activities, I may come under a relational duty to bear a fair share of its associated burdens.

You may worry my understanding of ‘relationship with the state’ is too loose. When I agree to follow a state’s laws, the relevant consensual relationship includes the state. This much is straightforward. But what about the relationship between democratic citizens? Or between drivers on the road? I think these, too, are relationships we have with the state, or an aspect of the state.

First, democratic citizens. Elections occur through a state’s institutions, like polling booths and electoral boards. And it is state institutions, like the legislature, which carry out a democratic mandate. For instance, a sweeping victory may confer a mandate upon the legislature to enact the victorious party’s platform. And even when the victory occurs through a referendum, it is ultimately state institutions which must enact the practical changes.

Second, drivers. To be sure, the primary participants are those who drive their vehicles on the road. But the state puts up signs and road markings. It maintains traffic lights. It operates a licensing scheme for qualified drivers. It hires police officers and traffic controllers. By travelling on the road, we position ourselves in a special way with this state-run activity. That position—the relation between travellers and those making safe travel possible—constitutes a relationship with an activity which forms an aspect of the state.^27^

### C. Legal Domains

If I am right, legal duties are partly constituted by special reasons. Those reasons apply within the limited domain of a particular relationship. Their presence makes moral duties into legal duties.

Put this way, this account takes us beyond, not just the state, but even bodies relatively analogous to the state. Any kind of relationship for which special reasons are applicable could be a law-having body. So far, I have focused on the state. Here, I am on solid footing: no one doubts the ability of states to have law. But we could go further. Municipal law is one type of law, but what about international law? Now, some doubt whether international law is really ‘law’. But we could go further still—what about canon law?

I happen to prefer a radically pluralist position. This explodes the kind of legal domains we could have. We may have special reasons arising from our relationship with the world community. Or even our church. Indeed, any relationship which gives rise to special reasons is, in principle, a body for which there is law. Consider friendships. Given their relationship, friends may have a duty to support each other. So, as it stands, friendship is a potential legal domain. The duties partly constituted by the special reasons of friendship form the ‘law of this friendship’.

To avoid this implication, we could insist only the state, or bodies sufficiently analogous to the state, can have law. Doing so requires an articulation of the state-like features which a law-having body must possess. Fleshing out those features is an available avenue to pursue for those who find a ‘law of this friendship’ especially unintuitive.^28^ For my part, however, I doubt the importance of this task. Suppose a mother, when addressing her child, refers to one of her family’s rules as the ‘law of her family’. Not much, I think, turns on whether she is mistaken.^29^

At this point, you might worry this indifference conflicts with a sentiment I expressed at the start. To motivate my account, I criticised views which sought to eliminate the line between legal duties and (simply) moral duties. Identifying which duties do, and which do not, form part of the law is a key feature of legal practice. And, if nothing else, this task presupposes that not every moral duty is a legal duty. A theory of law should seek to explain the social practice of law. Discriminating between legal and (simply) moral duties is just part of that practice. Failing to explain it comes at a serious cost.

But this sentiment is perfectly consistent with my present indifference. To reconcile them, consider the distinction between domain-specific and cross-domain assessments of what ‘law’ is. By domain-specific assessments, I mean whether a given duty counts as the law within a particular domain, like the state. This is what we refer to when we ask whether theft is illegal in England. By cross-domain assessments, I mean whether a particular domain, as compared to other domains, has a body which is capable of having law. This is what we refer to when we ask whether a neighbourhood association, as compared to the state, can have law.

To argue for the importance of distinguishing law from non-law, I relied on the nature of legal practice. The distinction forms an important part of the social practice which any theory of law should explain. This supports the significance of domain-specific assessments. Participants within legal practice—lawyers, judges and so on—spend much of their time asking whether a putative duty forms part of the law of their jurisdiction. Lawyers in England argue over, and judges answer, questions like the extent of the duty not to commit theft under English law. This is distinct from cross-domain assessments. From their perspective, whether it is mistaken to describe my family rules as ‘the law’ (in my family) is beside the point. What these lawyers care about is that my family’s rules do not form part of English law.

In short, legal practice is generally concerned with domain-specific assessments—that is, the legal status of duties *within their domain*. To be sure, some kinds of cross- domain assessments are relevant to legal practice. Some legal questions in a  particular jurisdiction turn on the legal status of a duty in a different jurisdiction. For instance, English judges may need to adjudicate disputes which involve a  choice-of-law provision referring to Spanish law as the source of applicable rules. Other cross-domain assessments, however, are largely irrelevant to legal practice. Take the question of whether a given domain is capable of having law at all. The status of Spain as a law-having body is not in doubt. The same cannot be said for the Sicilian Mafia. Whether criminal gangs have their own law might interest some philosophers, but it does not feature in the everyday practice of lawyers and judges—or even law professors and law students. My argument for the significance of distinguishing law from non-law, then, does not apply to this kind of cross- domain assessment.

## 6. Comparative Advantages

Why accept the constitutive reasons account? Here, I show it is preferable to the alternatives, for it directly addresses the problems with causal pedigree and judicial enforceability.

### A. Causal Pedigree

To start, I want to address a possible misunderstanding. Nothing about my account is inconsistent with the following claim: that acts of legal institutions always bear a causal relation to legal duties. This point is at the heart of Greenberg’s approach. But it is also consistent with my account. Take consent. Tourists may need to agree to abide by a state’s laws if they wish to enter the country. Giving permission to enter is an act by a state institution; that act forms part of the causal story for why the consent-based reason arises.

Given this, you may wonder whether my account collapses to causal pedigree. It does not. Suppose the relevant legal reason arises from a legislature’s democratic authority. On Greenberg’s view, what *explains* the duty’s legal status is its being caused by that legislature’s actions. On my view, however, we only need to know two facts: (i) that a democratically accountable institution made a decision; and (ii) that there is a special reason to respect the judgments of such institutions. Unlike Greenberg, I do not rely on *how* (i) led to (ii). Thus, my account does away with the need to specify a ‘legally proper way’ to cause legal duties to arise, as the relevant relationship between (i) and (ii) is constitutive, not causative. The duty is legal insofar as it is partly constituted by a legal reason.^30^

The primary problem with Greenberg’s account is overinclusiveness: it picks out too many duties as legal. Earlier, I focused on two examples: first, duties to resist the acts of legal institutions; and second, changes to the moral position which are only tenuously related to the acts of legal institutions. It would be costly for an account to count these duties as law. To be sure, Greenberg is aware of this problem. To address it, he says only changes caused in the legally proper way count as law. But this comes at the cost of explanatory power, for, as I argue, he lacks an attractive account of *why* these duties arise in a legally improper way. The constitutive reasons account fares better on both counts.

#### (i) Resisting evil

When we describe a state of affairs as evil, we ordinarily refer to reasons of universal applicability. Think of the killing of innocents, slavery and so on. Now, consider resistance to evil. Everyone has reason to prevent such evil from occurring, and we owe this to everyone else. So it is a general, not special, reason. This is why the duty to resist evil is not, without more, a legal duty.

Suppose the United States sought to create concentration camps. We have a duty to resist this. Now, suppose the United States does not exist. In this possible world, it is a gang of criminals who seek to enact this state of affairs. I nonetheless have precisely the same reasons to resist their activities. So the duty is insensitive to the existence of the United States. No legal reasons, special to our relationship to the United States, support it.

This, you may worry, is false. If the United States commits wrongs, Americans have a reason to protest—even if others do not. The thought is that a state’s actions are of greater concern to its citizens. Those citizens bear heightened responsibility for what their state does; that translates as a reason to stop it committing further wrongs. This could, for instance, take the form of complicity. On this view, Americans are complicit in what the United States does in their name. To not be complicit in the wrong, they must do things, like protest, to try and stop the United States from acting wrongly.

There is, however, a distinction between a reason and what that reason favours. The same reason can count in favour of different acts. The value of someone’s life is a reason for the doctor to take reasonable care when treating her—and a reason for me not to kill her. Giving incompetent medical care and committing murder are different acts, but the same reason—that her life is valuable—counts against it. Similarly, the same reason—that the United States has wronged others—may favour different acts. For Americans, it may require protests; for others, something else. The *reason*, however, applies to both. It is not limited to the relationship Americans have with the United States. To see this, let us return to complicity. The objection assumes the acts are wrong; if they are good, I should want to be ‘complicit’ in their occurrence. This is because complicity concerns the degree of responsibility one bears for an act. So it does not tell us anything about what favours, or disfavours, our action. Rather, it bears on the *strength* of our reasons, whatever they might be, given our situation. So complicity does not, alone, reveal a special reason—or, indeed, any reason. A further story is needed. And that story depends on general reasons. Our reason not to be complicit in murder is the value of the victim’s life. This is of general applicability. It is something we owe to the victim, not those with whom we are in a relationship of complicity. To be clear, this is entirely consistent with the thought that, with regard to a duty to protest, the relationship between American citizens and the United States matters. It is just that the relationship alters *what* that reason favours, rather than forming part of the *ground* of that reason. Reasons are only legal when the relationship matters in the latter sense.^31^

#### (ii) Visiting relatives

I should visit my relatives, given the valuable relationship I share with my family. Part of what makes that relationship are our duties to support one another. Given the loneliness caused by the lockdown, and the COVID pandemic generally, this duty may require me to visit my relatives. Now, the duty, to be sure, is composed of a special reason. I only owe the duty to my relatives, given my relationship with them. But my relationship is to *them*, not the state. So the duty does not form part of municipal law.

### B. Judicial Enforceability

The primary problem with Dworkin’s judicial enforceability account is underinclusiveness. Some moral duties are unenforceable in court, yet they are intuitively legal. Why insist on their legal status, despite their unenforceability? Because of the divergence between the reasons for the legal duty and the reasons for its unenforceability. A limitation period renders my claim unenforceable, but for reasons quite apart from those which underlie my legal rights. Perhaps the delay in bringing suit was faultless; I may be just as deserving of the right. Yet a court ought not to enforce it, so as to ensure legal certainty for a broad class of potential defendants.

Consider the following two scenarios. In Scenario A, I owe a moral duty to pay £500. It is also a legal duty as a matter of contract law. One reason for the duty is that it is good to keep my word. Another is that I benefit from the practice of contracting, which the state makes possible. Given these benefits, I have a fair-play reason to endure its burdens. Now consider Scenario B. Nothing about the reasons for the duty has changed. The only change concerns my identity. For I am no longer an ordinary person; I am, instead, a visiting sovereign of an independent nation. So there are good instrumental reasons against judicial enforcement of this duty. It would harm foreign relations, cause embarrassment and unsettle settled expectations between sovereigns. All this goes to why a court should not order me to pay. None of it, though, relates to whether I should pay. Of course I should. So the duty in Scenario A is precisely the same duty in Scenario B. It would therefore be odd if the duty in Scenario A, but not that in Scenario B, were legal.

My account vindicates this intuition. If the reasons for a duty stay the same, so too does its legal status. Returning to my promissory duty to pay £500, part of why I should pay the money is because I gave my word. This reason alone suffices to support a moral duty. But it is only a legal duty if we can identify a legal reason which underlies that duty. Here we can: the reason of fair play to endure reciprocal burdens.

But what if that reason went away? Not all promissory duties arise in the context of a co-operative activity which forms an aspect of the state. For instance, I could promise to pay £500 without intending to participate in the co-operative activity of contracting. If I did, the reason of fair play would not arise. Assume we are unable to identify another legal reason to support the duty. Even so, I might have a moral duty to pay the £500. The other reasons to keep my word, not affected by this change of circumstances, could well be decisive. But it would not be a legal duty.

## 7. Objections

To restate my view, the law of an entity, such as the state, consists of the duties partly composed of a reason arising from a relationship with that entity. Here, I address potential objections to this position—but not the objection that my account is underinclusive because it excludes legal duties which do not bind in morality. According to this worry, the law sometimes requires things we are morally free to refuse. My account of law cannot explain them, for I presuppose legal duties are always moral duties. Now, this is an important objection, but not one I can address here. It takes aim, not really at my particular account, but at anti-positivism more generally. The anti-positivist response, of course, is simply to reject the premise. It is to deny legal duties can be anything other than moral duties. Others have sought to motivate this denial.^32^ If more work is needed on this front, it must await another occasion. Given this, you can read this article as defending the following conditional claim: *if* anti-positivism is correct, we should focus on law’s constitutive reasons.

To that end, I respond to various objections. All share a common theme: they worry my account is overinclusive. More precisely, they identify moral duties which, at least intuitively, do not form part of the law.^33^ These duties fall into three groups: (i) those duties for which there is no judicial remedy; (ii) those arising from advice; and (iii) those which closely support state activities.

### A. Duties without Remedies

The constitutive reasons account allows for legal duties which are judicially unenforceable. Often this is intuitive. The government can act unlawfully even if the matter is non-justiciable before the courts. Similarly, a tortfeasor commits a legal wrong even after the limitation period runs out.

Sometimes, however, your intuitions might go the other way. Let us return to the contract law example. Suppose I promise to pay you £500 without securing an agreement to receive anything in exchange. Plausibly, my promise suffices to ground a duty to pay. Say I refuse. I thereby wrong you. Yet you might be unable to get a court to enforce that duty. In England, damages are typically only available when the promise is backed by good consideration.

So long as the promise was intended to create a legal relation, I have said a legal reason supports the promissory duty. This is because it partly consists of the fair play reason to endure reciprocal burdens arising from active participation in a beneficial activity. This suffices to make the promissory duty legal; no further requirement of consideration is necessary. I am therefore committed to saying gratuitous promises can impose legal duties.

This is entirely consistent with insisting upon consideration before awarding damages. Here are two reasons the law might restrict remedies in this way. First, consideration could be a useful formality. It evidences an intention to create legal relations, ensures the robustness of that intention by performing a cautionary role and offers a way to express that intention in a characteristic way.^34^ Second, we might say the breach of a duty arising from a gratuitous promise is wrong, but not so wrong as to warrant a coercive remedy. For that, we may need the particular unfairness of resiling from a bargain.^35^

Perhaps you are unsatisfied, as we are still left with the following picture: gratuitous promises ground legal duties, albeit unenforceable ones. Why not just say they fail to ground any legal duties? Here is why: because my view better explains the way the doctrine of consideration is perceived. In *White v Jones*, for instance, the UK House of Lords developed a doctrine to extend a remedy for some breaches of gratuitous promises.^36^ There, Lord Goff accepted that this extends to the plaintiff ‘what is, in substance, a contractual cause of action’.^37^ The impulse to develop the law in this way is readily explicable if we view consideration as an external restriction on the availability of a remedy, rather than as a core feature of the underlying duty. Nor is *White v Jones* unique. The law often struggles to decide whether to enforce gratuitous promises. Thus, ‘the law would be rendered more intelligible and clear if the need for consideration were abolished’.^38^ This is an internal, doctrinal critique of the law. It accuses consideration of frustrating the development of an intelligible principle by which to explain the substantive law of contract. Again, this is consistent with the thought that consideration is a remedial restriction.

More generally, the doctrine of consideration has inspired particular hostility.^39^ At first glance, this is puzzling. Not all moral duties form part of the law. Nor is there widespread unease about this. But it is altogether more understandable if we suppose gratuitous promises impose legal duties. We might think, for example, that the law should, as a starting point, enforce *legal* duties. Sometimes there are good reasons not to. Think of justiciability doctrines. Even so, the lack of enforcement might leave us uneasy; at a minimum, we require a powerful justification for non-justiciability. The same dynamic could explain the particular unease with consideration in contract law.

### B. Advisory Duties

In the midst of the COVID pandemic, the UK government sought to alter the behaviour of its residents. Often it did so not by proposing legislation or issuing regulations, but by providing advice. These statements did not go through a formal process of approval. And they were expressed in non-imperative terms.

Nonetheless, it is plausible that some residents came under a moral duty to do what the government advised. Suppose the government advised us to stay home. Perhaps we ought to stay home anyway, to protect the health of others. This alone could not, under my account, make the duty legal, for the reason to protect the health of others is general, not special. But other reasons could support this duty, too. For instance, it might be unfair to benefit from the sacrifices of others, while disregarding the corresponding burdens. Since lockdowns were an intimate aspect of the state, my account identifies this reciprocity-based reason as a legal reason. So we would have a legal duty to stay home. In some ways, this captures the experience on the ground. At the time, many, including officials, treated advice in just the same way as regulatory rules. To be clear, this is not to say officials were justified in doing so. I reject the conflation of a duty’s legal status with its enforceability. Nonetheless, you might find my conclusion—that advice sometimes led to legal duties—unintuitive for two reasons: first, because the advice did not go through the formal processes which are characteristic of law; and second, because the government did not intend for its advice to be law. Here I respond to both.

#### (i) Formal process

For many, the line between ‘law’ and ‘advice’ during the COVID pandemic was indistinguishable. The UK government chose to institute its COVID strategy through a complicated mix of regulatory and advisory rules. Often, it was difficult to discern where one began and the other ended. Nor, indeed, did the distinction matter much to ordinary residents. Their lives were significantly altered regardless. Further, officials, predictably, had trouble telling the regulatory and advisory rules apart. This led to confusion about which duties were enforceable and which were not. And that posed a serious challenge to the rule of law.

Given this, it may have been better for such advice to go through a formal, characteristically legal process. That the government failed to do this might motivate you to deny that the advice counted as law. My account offers a different explanation. It allows for a powerful criticism of government-by-advice, not because it fails to be law, but because it *is* law. Precisely because the advice led to legal duties, it should have gone through the formal process characteristic of law. And precisely because the advice was law, the lack of certain procedural safeguards was regrettable, for the rule of law attends to the harms which might otherwise arise from law.^40^ Perhaps the issuance of advice was necessary, but it may have led to the very harms the rule of law guards against.

#### (ii) Intent

Another worry is that the government did not intend to create law. Not all law, however, is formed intentionally. Some legal rules are customary—and those rules arise from social practices which do not always aim to construct law. Similarly, the government may have unintentionally created legal duties via advice. A related worry is that the government intended to offer suggestions, not orders. Now, in the context of COVID lockdowns, this is doubtful. There, the government may well have intended to create mandatory duties, even while framing them as ‘advice’. In any event, some of its advice, during the exceptional context of the pandemic, led to moral duties. So we know the government’s acts diverged from its intent. The question is simply how far that divergence went.

### C. Ancillary Duties

Here, I focus on two contexts: constitutional conventions and elections. I describe their associated duties as ‘ancillary’ because they relate to the functioning of core state institutions. Constitutional conventions play a key role in regulating how our constitutional actors behave. And elections allow legislatures to enjoy democratic legitimacy.

#### (i) Constitutional conventions

To explain how constitutional conventions might bind, Jaconelli argues that they are supported by a reason arising from mutual benefit and burden.^41^ Some constitutional conventions restrain the ruling party. So far as power periodically changes hands, these restraints will eventually apply to the opposition party, too. It may be beneficial, to all parties, to accept these restraints. Although burdensome to the party in power, it confers valuable protection when that party is in opposition. These political parties, insofar as they benefit from such restraints, have reason to accept their respective burdens. I have described such reasons as arising from fair play. Under my account, the duties to which constitutional conventions refer, insofar as they are supported by such reasons, are legal duties. This presents a challenge, for we typically think of constitutional conventions as rules which, although they form part of the constitution, are not law.

What motivates this thought? Dicey offers one argument: constitutional conventions cannot be law since they are neither ‘enforced or recognised by courts’.^42^ Munro offers another: that, in contrast to the systemic nature of law, conventions form a ‘discrete unconnected set’.^43^ The truth of both propositions is doubtful, even on their own terms.^44^ First, the claim about courts. As a descriptive matter, courts often recognise the existence of constitutional conventions,^45^ and perhaps they sometimes even enforce them, too.^46^ Second, the claim about systematicity. Some constitutional conventions are intentionally created by actors. Those actors, in turn, are authorised to do so by a separate, power-conferring constitutional convention. If so, these conventions would bear the systemic relation between primary and secondary rules which Hart thought was the mark of law.^47^

For most, I suspect it is the claim about judicial enforceability which motivates the denial of legal status to constitutional conventions. That is, you might descriptively accept that some courts enforce conventions. You could, however, normatively reject the appropriateness of such enforcement. And that distinguishes conventions from law. But the distinction remains undermotivated, for, as we saw, not all laws are enforced in court.^48^ Since some laws are not legally enforceable, the presence of enforceability cannot distinguish  conventions from law.

This, you may worry, goes too far. Perhaps not every law is judicially enforceable. But judges plausibly have a defeasible duty to apply any legal duty before them. If they do have such a duty, the starting point, for legal duties, is judicial enforceability. The objection would be that constitutional conventions, by contrast, lack this starting point. Put this way, however, the point misleads. True, for most legal duties, this defeasible duty will likely be decisive. By contrast, most constitutional conventions will likely attract serious concerns of judicial enforceability. The force of this objection therefore relies on the contrast between law, understood as a distinct category, and conventions. But it is the extent of the former category—the set of legal duties—which we are trying to discern. And the difference dissipates once we investigate a subset of those legal duties. Consider duties which, although obviously legal, are intimately connected to the political choices of elected officials. The judicial enforcement of these duties, just as much as constitutional conventions, raise serious concerns.

#### (ii) Voting

There is no legal duty to vote in the United States, but such a legal duty exists in Australia. The question is whether my account can make sense of this difference. One possibility is to deny that American citizens are morally obligated to vote. On this view, legislative intervention is needed for a moral duty to arise, like in Australia.

Many, however, think a moral duty exists in both jurisdictions. So we must now turn to the constitutive elements of this moral duty. In Australia, one of the reasons underlying the duty is the legislative determination in favour of compulsory voting. Hence, we can identify a legal reason—to respect democratic decisions. This explains why Australia has a legal duty to vote. But can we explain why America does not?

To do so, I must show that no legal reasons bear on the moral duty of Americans to vote. This looks like a significant challenge, for there is an obvious possibility. Political participation could be valuable. That value arises from our relationship with the polity. Given this value, Americans could have a reason to vote. This reason, under my account, looks like a legal reason.

On one view, this value arises no matter our preferred candidate. What matters is our participation in the electoral process. Now, such a value is easy to grasp in small, homogeneous, discursive societies—think of an idealised version of ancient Athens. There, we might think elections facilitate valuable forms of deliberation and civic friendship. But you may doubt this wholly participatory value arises in modern pluralistic societies, for the mere act of voting, taken alone, is rather thin. And even if it exists, you may doubt this value is sufficiently strong to contribute to a *duty* to vote.

Given this, you may seek to enrich the value by reference to our preferred candidates. This allows for a more promising approach, for electoral outcomes can have important consequences. So we may have moral reason to vote for, and otherwise assist, the better candidate. There is reason to help good candidates win public office. But this, in isolation, looks like a general, not special, reason. We should support people who will allow for good consequences and oppose others who will cause harm. This is a reason we always have, however it is not a special reason. So it cannot be a legal reason.

In response, you could turn to fair play.^49^ By voting, we can prevent injustice and achieve other beneficial consequences. We all benefit from this. By refusing to vote, we free ride on those who did vote to prevent injustice. Voting to achieve such ends is a co-operative enterprise. It requires each participant to endure the burden of casting a vote. As a matter of reciprocity, we should do our fair share to contribute to the common benefit of good electoral outcomes.

Nonetheless, here are a few arguments against the existence of fair play reasons to participate in elections. First, we may doubt the electoral process, as a whole, is a co-operative enterprise, for it is irreducibly competitive. Elections are how duelling factions seek political victory over one another. When you and I vote for diametrically opposed political candidates, our shared activity is not co-operative. By contrast, it is easier to see how political parties, who organise to seek the victory of their preferred candidates, are engaged in a co-operative enterprise. But the activities of a particular political party are distinct from the state. Second, we may doubt whether a person who refuses to vote meaningfully participates in the electoral activity. Earlier, I suggested fair play reasons only arise for those who participate in the co-operative enterprise. Such a restriction is one way to address Nozick’s objection concerning the neighbourhood association which supplies entertainment. If so, this decisively cuts against an electoral bystander having a reason to vote. To be sure, there are ways, aside from voting, to participate in the electoral process. For example, I could volunteer at the polls or be an activist. But then I am enduring a burden, and therefore the question of free riding would not arise.

## 8. Conclusion

Suppose the law consists of a duty to pay half my income in taxes. For positivists, this bears no necessary relation to there being a moral duty to do so. For anti- positivists, by contrast, I must *necessarily* have a moral duty to pay that amount.

Now take the language of necessity away. The question, suitably revised, is whether, under the law, there exists a moral duty to pay half my income. This issue attracts the attention of not just legal philosophers, but political theorists too. A long line of thought, travelling under the familiar label of ‘political obligation’, evaluates the prospects of various considerations which might support a duty to pay. These considerations include consent, democracy and fair play—precisely the reasons I identify as legal reasons.

Such discussions, however, often assume a deeply positivistic outlook. First they ask what the law is; then they ask whether a duty, apart from the merits of that law, exists. The constitutive reasons account flips this order. It first looks to the considerations which underlie the duties we have. Only then does it ask what the law is.

Even those who disagree with my account of legal reasons can accept this approach. You can add, jettison or modify the set of reasons which are legal reasons. What matters is that we agree on the significance of the reasons which underlie legal duties. To explain legal duties, we should look to the nature of those reasons.

A particular kind of reason—the legal reasons—makes moral duties into legal duties.

## Footnotes

1 I will generally talk of duties, but I mean for my arguments to apply to privileges, powers and so on. I will be more specific when it matters.

2 Such scepticism travels under the label of eliminativism. The trouble is the label masks the diversity of views it describes, for there are some important differences in what those views seek to eliminate. As a preliminary matter, the label could refer to the elimination of a distinctively legal *normativity*, ie the sense in which a legal duty could bind without being morally binding: see Scott Hershovitz, ‘The End of Jurisprudence’ (2015) 124 Yale LJ 1160, 1193. But this version of eliminativism is just another way to describe anti-positivism. So let us put that view to one side. There remains a range of different possible eliminativist views. We could imagine a view which denies the existence of a discrete category of legal duties, even if that category forms part of a broader class of moral duties. Call this category eliminativism. Another view seeks to eliminate talk of law in legal practice: Lewis A Kornhauser, ‘Doing Without the Concept of Law’ (2015) NYU School of Law Public Law and Legal Theory Research Paper Series Working Paper 15-33. Call this discourse eliminativism. Still others are sceptical of whether the concept of law has an essential nature: Hilary Nye, ‘Does Law “Exist”? Eliminativism in Legal Philosophy’ (2022) 15 Washington University Jurisprudence Review 29. Call this concept eliminativism. Only category eliminativism—the denial of a discrete set of legal duties—poses a challenge, for my account is entirely consistent with the thought that, as a practical matter, it would be better for lawyers to revise their practices to avoid talk of law (ie discourse eliminativism). I am concerned with the practices we have, not whether or how they should be changed. It is also consistent with the thought that the concept of law—understood as an idea which picks out some practices as law—lacks a single nature (ie concept eliminativism). Such concepts of law are what Dworkin described as ‘sociological’: Ronald Dworkin, *Justice in Robes* (Harvard UP 2006) 2–4. However, my concern is with the grounds of legal propositions, not the essential features of what makes some social structures count as a legal system: cf Ronald Dworkin, *Law’s Empire* (Harvard UP 1986) 4.

3 To be clear, this is not meant as a knock-down argument against category eliminativism, though I do think it struggles with this feature of legal practice. For instance, a common eliminativist strategy is to disambiguate ‘the law’ into, among other things, genuine moral duties and predictions of what officials are likely to do. If so, ‘the law’ would be a disjunctive combination of these two possibilities (among others). One problem with this view is its inability to account for the thought, internal to legal practice, that predictive claims just are not law. But I will not pursue that line here, for there is a more serious problem. Lawyers make claims about legal content all the time, and it is implausible to suppose they *ever* mean to refer to an entirely open set of moral duties. Nobody thinks the law consists of all genuine moral duties. So a further disambiguation is required. You could read this article as defending a possible way to achieve this further disambiguation. That is, on those occasions when ‘the law’ refers to genuine moral duties, it refers only to those duties picked out by my account.

4 Mark Greenberg, ‘The Moral Impact Theory of Law’ (2014) 123 Yale LJ 1288, 1320–3.

5 Ronald Dworkin, *Justice for Hedgehogs* (Harvard UP 2011) 404–6. I say ‘on some readings’ given an interpretive disagreement. The difference lies in where Dworkin stands in relation to the causal pedigree thesis. According to that thesis, all legal rights are causally traceable to the acts of political institutions. This corresponds to two possible readings of Dworkin. The requirement for judicial enforceability could be in *addition* to causal pedigree: see Nicos Stavropoulos, ‘Why Principles?’ (2007) Oxford Legal Studies Research Paper No 28. This reads Dworkin as proposing a possible way to flesh out the moral impact theory. If this reading is correct, Dworkin would only disagree with Greenberg to the extent the latter assumes that causal pedigree suffices for a duty’s inclusion in law. Alternatively, the requirement of judicial enforceability could be a *substitute* for causal pedigree: Greenberg, ‘Moral Impact Theory’ (n 4) 1299–30, fn 18. On this reading, Dworkin and Greenberg fundamentally disagree. None of my core arguments turn on this interpretive disagreement; I leave open which reading best reflects Dworkin’s thinking.

6 Strictly speaking, a claim about what should occur is not future-tensed. That a court should enforce a right is both a claim about what should occur tomorrow and what should have occurred yesterday. When I say Dworkin looks to the future, I mean a duty’s enforcement can only arise, as a temporal matter, *after* that duty comes into existence. Conversely, the way a duty is caused comes temporally *prior* to that duty’s existence, leaving to one side the possibility of backwards causation.

7 This is a claim about the nature of legal duties. It tries to explain why, among our moral duties, some (but only some) form part of the law of a given jurisdiction. Here, I want to emphasise the limits of this claim. I do not purport to offer a full theory of law. I will not address borderline instances of legal systems. I will not seek to explain why some societies have law, while others do not. My focus, rather, is on jurisdictions which uncontroversially have law, like the United States. What I want to explain is *why* some moral duties form part of American law, as distinct from the plethora of other moral duties which obtain within the United States.

8 Greenberg, ‘Moral Impact Theory’ (n 4) 1321–3.

9 I do not mean to suggest the problem I sketch is the *only* problem with Greenberg’s account of a ‘legally proper way’. For other issues, see Hasan Dindjer, ‘The New Legal Anti-Positivism’ (2020) 26 Legal Theory 181, 202–4.

10 Mark Greenberg, ‘The Moral Impact Theory, the Dependence View, and Natural Law’ in George Duke and Robert P George (eds), *The Cambridge Companion to Natural Law Jurisprudence* (CUP 2017) 275, 289–91. To motivate this disavowal, Greenberg offers this example. Suppose the legislature enacts a statute. It clearly designates a particular scheme. Call this scheme A. But things go awry. For whatever reason, the legislature fails to cause a convergent expectation on scheme A. Perhaps this is because a critical player accidently misinterprets the statute, or because of a pervasive psychological bias, or so on. In any event, the population converges on scheme B, not A. So it is scheme B which becomes morally salient. We might then have a moral duty to participate in scheme B. And that, Greenberg says, is a *legal* duty. Yet the legislature, on any plausible view of intention, sought to require scheme A. And the statute, on any plausible view of linguistic meaning, requires scheme A.

11 Greenberg, ‘Dependence View’ (n 10) 281–2.

12 Schaus raises the same worry in Steven Schaus, ‘How to Think About Law as Morality: A Comment on Greenberg and Hershovitz’ (2015) 124 Yale LJ Forum 224, 234.

13 Hasan Dindjer largely anticipates these arguments: Dindjer (n 9) 204–8.

14 As Sager points out, this occurs both when legislatures wield a power which is non-justiciable in the courts, and when a constitution confers a non-justiciable right to socioeconomic goods: Lawrence Sager, ‘Fair Measure: The Legal Status of Underenforced Constitutional Norms’ (1978) 91 Harv L Rev 1212; Lawrence Sager, ‘Material Rights, Underenforcement, and the Adjudication Thesis’ (2010) 90 BUL Rev 579.

15 *R v Withers* [1975] AC 842, 854 (Lord Reid), 860 (Viscount Dilhorne), 863 (Lord Simon), 877 (Lord Kilbrandon).

16 For the avoidance of doubt, I think legal reasons must be moral reasons. True, for a reason to be legal, something must distinguish it from all the other reasons. But that distinction need not lie between moral and non-moral reasons. It may, instead, be a distinction between moral reasons.

17 Joseph Raz, *Ethics in the Public Domain* (OUP 1994) 342–3.

18 What about entirely trivial reasons? By this I mean reasons which play no part in the explanation of the content of the duty. This occurs when the legal reason makes no difference to what the duty requires. When this is the case, removing the reason does not alter the scope of the duty in any way; the duty prohibits precisely the same set of acts as before. Such reasons fail to *compose* the relevant duty. It therefore cannot make that duty a legal duty. This is an issue of composition: when reasons can properly be said to partly compose the duty. I cannot give a complete answer to this mereological issue here, but there is one possibility I find especially promising. A reason composes a duty when it forms a necessary aspect of a set of reasons, which together suffice to ground the precise content of that legal duty. This bears some similarity to a leading account of causation: see Richard Wright, ‘Causation in Tort Law’ (1985) 73 Cal L Rev 1735, 1788–1803. Yet I say, unlike Greenberg, that my account is non-causal. What gives? Notice how, on my account, the determination is not something we would typically describe as causal. It is not the relation between an event and a state of affairs. It is, rather, a relation between duties and the reasons that underlie them. This is metaphysical, not causal, determination. Both, however, are one-directional determination relations (ie X grounds/causes Y, but not the other way around). So it is unsurprising that similar ideas apply across both domains.

19 Recall that, on my account, a duty is legal so far as *one* legal reason (like consent, democracy or fair play) composes the duty. Why is this important? Because any given legal reason, taken in isolation, cannot plausibly explain the entire content of the law. Consider consent. The effort to explain legal duties as consensual is subject to well-known difficulties. The most pressing is that many people do not, in fact, consent to the rule of the state: see John A Simmons, *Moral Principles and Political Obligations* (Princeton UP 1979) 83–100; Ronald Dworkin, *Law’s Empire* (Harvard UP 1986) 192–3. Nonetheless, consent can still help explain the legal duties of officials (who choose to stay in office) and short-term tourists (who choose to visit).

20 HLA Hart, ‘Are There Any Natural Rights’ (1955) 64 Philosophical Review 175, 185; John Rawls, ‘Legal Obligation and the Duty of Fair Play’ in Sidney Hook (ed), *Law and Philosophy* (NYU Press 1964) 9–10.

21 Robert Nozick, *Anarchy, State, and Utopia* (Basic Books 1974) 90–5.

22 For a similar argument, see George Klosko, *The Principle of Fairness and Political Obligation* (Rowman & Littlefield 2004) 38–9.

23 Richard Dagger, *Civic Virtues: Rights, Citizenship, and Republican Liberalism* (OUP 1997) 69–70.

24 As applied to law, it is tempting to look to the state itself as the co-operative enterprise. The continued operation of the state benefits us all, but it also requires us to endure certain burdens. But this is a mistake. By setting our sights lower, we can place the legal reasons arising from fair play on more solid footing. Rather than consider the state as a whole, we could turn to the more particular activities with which the state is intimately involved.

25 In this way, my account of legal duties relies on the moral distinction between general and special reasons. Plausibly, this distinction reflects a fundamental feature of our normative reality. It reflects a real difference in our reasons; it is not just a conceptual apparatus we have chosen to construct. If I am right, this should go some way to address the eliminativist worry that there is nothing ‘real’ about the distinction between legal duties and non-legal moral duties.

26 Hence, short-term tourists may have a consensual duty to comply with the laws of their hosts. To vacation in France, I may need to agree to abide by French laws, even those which strike me as seriously misguided. This gives me a special reason to conform to French law. To be sure, when I travel to France, I do not plausibly agree to comply with all the moral duties which obtain in France. My agreement is only directed at a subset of those moral duties: the duties which form French law. This presupposes an account of French law. So you may worry this leads to a circularity problem. But there is a straightforward solution. The set of duties I accept are (for the most part) just those legal duties which apply, in non-consensual fashion, to French residents. To identify them, we must now turn to the special reasons which apply to French citizens, given their relationship to the French state. Doing so helps us see which duties tourists agree to follow.

27 At this point, you may want an exhaustive account of which activities form an aspect of the state and which do not. That requires a theory of the state, which this article lacks. I do, however, want to insist on two points. First, we *should* need a theory of the state to arrive at a full picture of what constitutes the law of a given state. To fully account for American law, we need to know: (i) what law is; and (ii) what counts as part of the United States. Here, I have primarily focused on (i). Second, my account is broadly consistent with an intuitive sketch of what does, and what does not, form an aspect of the state. For instance, the relationship among citizens in a democracy forms part of the state. Hence, the special reasons Americans have to respect democratic decisions make certain moral duties part of American law. By contrast, my relationship with a friend does not form part of the state. This is why, although I have special reasons to support my friends, the duties those reasons support are not thereby part of American law.

28 As such, you can read the article as advancing the following, incomplete claim: legal duties are moral duties partly composed by a reason arising from a relationship with the state, or bodies sufficiently analogous to the state. This is incomplete insofar as I leave open-ended the criteria which might render a body sufficiently analogous to the state.

29 Again, this might be wrong-headed. But I find the promiscuity of my approach to be a feature, not a bug. It encompasses law-talk in a variety of domains, while preserving the intuition that the line between law and non-law is important within a given domain. In other words, it is deflationist about the significance of cross-domain assessments, while rejecting eliminativism about domain-specific assessments.

30 To be sure, this reason might only arise as a causal consequence of a legal institution’s act. So the existence of the constitutive reason covaries with the existence of a causal relation between a legal institution’s act and the moral situation. But my account does not depend on that causal story. An obligation’s causal pedigree plays no part in my explanation of why it forms part of the law.

31 You could press the objection further. Suppose my friend is about to commit theft. The value of our friendship, you may say, gives me a reason to stop her. It is distinct from the general reason to stop strangers from committing wrongs. The interests of the potential victim ground a general reason to prevent theft. We may also think, however, that the value of my friendship gives me a special reason. It only applies within my friendship. I owe it not only to the victim, but also to my friend to stop her from stealing. Similarly, I may owe it to the United States, given our relationship, to stop it from committing wrongs. This special reason, however, is parasitic on a general reason. Even on this view, I only owe it to the United States to stop it from *wronging* others. That an act is wrong is therefore ineliminable from the explanation of why Americans in particular should intervene. And the wrongness of the act is explained by reasons quite apart from their relationship with the United States. Any reason which favours a duty to protest must include a general consideration. So it can, at most, be a composite reason: a reason constituted by both general and special considerations. Such composite reasons are not (wholly) special reasons. And only special reasons can be legal reasons.

32 Dworkin, *Justice for Hedgehogs* (n 5) ch 19; Mark Greenberg, ‘How Facts Make Law’ (2004) 10 Legal Theory 157.

33 Notice something important. Overinclusiveness objections are unavailable to those sympathetic to category eliminativism, for they take the following form: (P1) There is a set of duties which form the content of the law. (P2) If my account is true, a given duty forms part of that set. (P3) That duty does not form part of that set. (C) My account is therefore wrong. Category eliminativism gets off at P1. They do not accept the existence of a discrete set of legal duties. But the overinclusiveness objection needs P1, for it assumes the law has a certain content, which my account overshoots. I take such objections seriously, because I accept there is a genuine sense in which some duties are not legal. I just think these objections fail on the merits. By contrast, category eliminativism cannot accept these objections as meaningful. That is because it denies the ‘set of legal duties’ has a true referent. Hence, there is nothing for an account to be overinclusive of. Say you agree these objections are meaningful, irrespective of whether they succeed or fail. This is yet another reason to reject category eliminativism.

34 Lon L Fuller, ‘Consideration and Form’ (1941) Colum L Rev 799, 800–3.

35 This is because there might be countervailing reasons which count against coercive enforcement: see Mindy Chen-Wishart, ‘In Defence of Consideration’ (2013) 13 OUCLJ 209, 213–14.

36 *White v Jones* [1995] 2 AC 207.

37 ibid 264.

38 Andrew Burrows, *Understanding the Law of Obligations* (Hart Publishing 1998) 164, 197.

39 See eg Wright, ‘Ought the Doctrine of Consideration to Be Abolished from the Common Law?’ (1936) 49 Harv L Rev 1225.

40 See Joseph Raz, *The Authority of Law* (OUP 1979) 221–2.

41 Joseph Jaconelli, ‘Do Constitutional Conventions Bind?’ (2005) 64 CLJ 149, 171–2.

42 AV Dicey, *Introduction to the Law of the Constitution* (10th edn, Macmillan 1964) 417.

43 Colin Munro, ‘Laws and Conventions Distinguished’ (1975) 91 LQR 218, 233.

44 NW Barber, ‘Laws and Constitutional Conventions’ (2009) 125 LQR 294, 309.

45 Geoffrey Marshall, *Constitutional Conventions: The Rules and Forms of Political Accountability* (OUP 1984) 13–15; NW Barber, *The Constitutional State* (OUP 2010) 40.

46 TRS Allan, *Law, Liberty, and Justice: The Legal Foundations of British Constitutionalism* (OUP 1993) 244; Ivor Jennings, *The Law and the Constitution* (1959) 177; *Copyright Owners Reproduction Society Ltd v EMI* [1958] 100 CLR 597 (HCA). Whether the courts should enforce these conventions is another matter: see Timothy Endicott, ‘Making Constitutional Principles into Laws’ (2020) 136 LQR 175, 178.

47 Adam Perry and Adam Tucker, ‘Top-Down Constitutional Conventions’ (2018) 81 MLR 765, 776–9.

48 Maley makes this precise point in the context of constitutional conventions: William Maley, ‘Laws and Conventions Revisited’ (1985) 48 MLR 121, 128.

49 eg Julia Maskivker, *The Duty to Vote* (OUP 2019) 64.

